# Laparoscopy-Assisted versus Open Hepatectomy for Live Liver Donor: Systematic Review and Meta-Analysis

**DOI:** 10.1155/2017/2956749

**Published:** 2017-11-07

**Authors:** Bin Zhang, Yu Pan, Ke Chen, Hendi Maher, Ming-Yu Chen, He-Pan Zhu, Yi-Bin Zhu, Yi Dai, Jiang Chen, Xiu-jun Cai

**Affiliations:** ^1^Department of General Surgery, Sir Run Run Shaw Hospital, School of Medicine, Zhejiang University, 3 East Qingchun Road, Hangzhou, Zhejiang Province 310016, China; ^2^School of Medicine, Zhejiang University, 866 Yuhangtang Road, Hangzhou, Zhejiang Province 310058, China

## Abstract

**Objective:**

To assess the feasibility, safety, and potential benefits of laparoscopy-assisted living donor hepatectomy (LADH) in comparison with open living donor hepatectomy (ODH) for liver transplantation.

**Background:**

LADH is becoming increasingly common for living donor liver transplant around the world. We aim to determine the efficacy of LADH and compare it with ODH.

**Methods:**

A systematic search on PubMed, Embase, Cochrane Library, and Web of Science was conducted in May 2017.

**Results:**

Nine studies were suitable for this analysis, involving 979 patients. LADH seemed to be associated with increased operation time (WMD = 24.85 min; 95% CI: −3.01~52.78, *P* = 0.08), less intraoperative blood loss (WMD = −59.92 ml; 95% CI: −94.58~−25.27, *P* = 0.0007), similar hospital stays (WMD = −0.47 d; 95% CI: −1.78~0.83, *P* = 0.47), less postoperative complications (RR = 0.70, 95% CI: 0.51~0.94, *P* = 0.02), less analgesic use (SMD = −0.22; 95% CI: −0.44~−0.11, *P* = 0.04), similar transfusion rates (RR = 0.82; 95% CI: 0.24~3.12, *P* = 0.82), and similar graft weights (WMD = 7.31 g; 95% CI: −23.45~38.07, *P* = 0.64).

**Conclusion:**

Our results indicate that LADH is a safe and effective technique and, when compared to ODH.

## 1. Introduction

Liver transplantation from living donors is a potential treatment for end-stage liver disease. And due, in part, to the limited number of available livers from deceased patients, living donor liver transplantation (LDLT) has become an established solution. Since the first successful LDLT for a child in 1989 [1], this life-saving procedure has developed rapidly, providing similar or even better outcomes, especially in children, in comparison with cadaver liver grafts [2]. Living donors are typically healthy adults; therefore the donor's safety is paramount.

Over the past two decades, laparoscopic surgery has been widely applied to liver surgery. In 2002, Cherqui et al. [3] reported the first case of laparoscopic living donor left lobectomy and laparoscopic LDLT was increasingly used in some centers. However, owing to technical difficulties, this procedure developed relatively slowly. The first case of laparoscopic-assisted hybrid living donor hepatectomy (LADH) was reported by Koffron et al. [4] in 2006, in which hands were introduced into the abdomen while still maintaining the pneumoperitoneum. In this procedure, a laparoscopic technique is employed for mobilization of liver and hilar dissection; however, the parenchymal transection is performed as an open procedure. As a result, this hybrid procedure achieved the advantage of avoiding a large subcostal incision while retaining the safety and familiarity of an open dissection and resection. In addition, laparoscopic-assisted surgeries offered surgeons an opportunity to accumulate expertise before converting to complete laparoscopic living donor hepatectomies.

Several studies have compared the outcome of laparoscopic-assisted living donor hepatectomy (LADH) with widely used open living donor hepatectomy (ODH). However, no consensus has been reached on this topic; it is still not clear which method is of more benefit to the donor. In this setting, we comprehensively collected relevant data and conducted a systematic review with meta-analysis to assess the feasibility, safety, and potential benefits of laparoscopic-assisted living donor hepatectomy.

## 2. Materials and Methods

### 2.1. Systematic Literature Search

This meta-analysis was finished by searching electronic databases of PubMed, Embase, Cochrane Library, and Web of Science and scanning reference lists of articles in* May 2017* by Two investigators (B. Zhang and Y. Pan) independently. Strategies included the terms “laparoscopy”, “laparoscopic”, “minimally invasive”, “hybrid”, “hand-assisted”, “hepatectomy”, “liver resection”, “hepatic resection”, “living donor”, and “liver donor”. All eligible studies in English were retrieved, and their bibliographies were checked for potential relevant publications.

### 2.2. Eligibility Criteria

Studies comparing laparoscopy-assisted and open living liver donor hepatectomy are included for the systematic review and meta-analysis including prospective or retrospective case series. Studies were excluded if they met any of the following criteria: (1) case reports, letters, reviews, editorials, and studies lacking control groups; (2) studies that did not report the type of surgery or operation data; (3) if dual (or multiple) studies were reported by the same institution and/or authors, only the most recent publication or the highest quality of studies was included. However, articles from the same authors or centers but with different patient cohorts were included.

### 2.3. Data Extraction and Quality Assessment

Two investigators (M. Y. Chen and H. P. Zhu) independently assessed publications for inclusion and extracted data from eligible studies, including the baseline characteristics, such as first author, publication year, country of region, study type, sample size, and operation outcomes (operation time and intraoperative estimated blood loss) and postoperative outcomes (overall complications and length of hospital stay). The primary outcomes of the study include blood loss, complications, and analgesic use. The secondary outcomes are operation time, transfusion, length of stay, and graft weights. We made attempts to contact corresponding authors for missing data points. Only one author provided requested data for analysis [5].

The quality of the researches was evaluated by The Newcastle-Ottawa Quality Assessment Scale (NOS). The scale ranged from 0 to 9 stars: studies achieving more than or equal to 6 are deemed as good methodologically.

### 2.4. Statistical Analysis

All analyses were performed with Review Manager Version 5.3 (The Cochrane Collaboration, Oxford, United Kingdom). Risk ratio (RR) with a 95% confidence interval (CI) was used for the comparison analysis of dichotomous variables. The same continuous parameters were expressed as weighted mean difference (WMD) in the same unit or standard mean difference (SMD) for different unit with 95% CI. When data in individual studies was presented as median and a range, the means and standard deviations (SDs) were estimated by Hozo et al. [6]. The test of heterogeneity, which indicated between-study variance, was evaluated according to Cochran's test and Higgins-squared statistic [7]. Pooled effects were calculated using a random-effects model, unless heterogeneity was less than 50% or *P* < 0.05. Graphical funnel plots were generated to determine visual inspections for publication bias.

We conduct subgroup analyses in the studies focusing on right lobe hepatectomies (RH) and left lobe hepatectomies (LH).

## 3. Results

### 3.1. Study Eligibility

**Figure 1:**
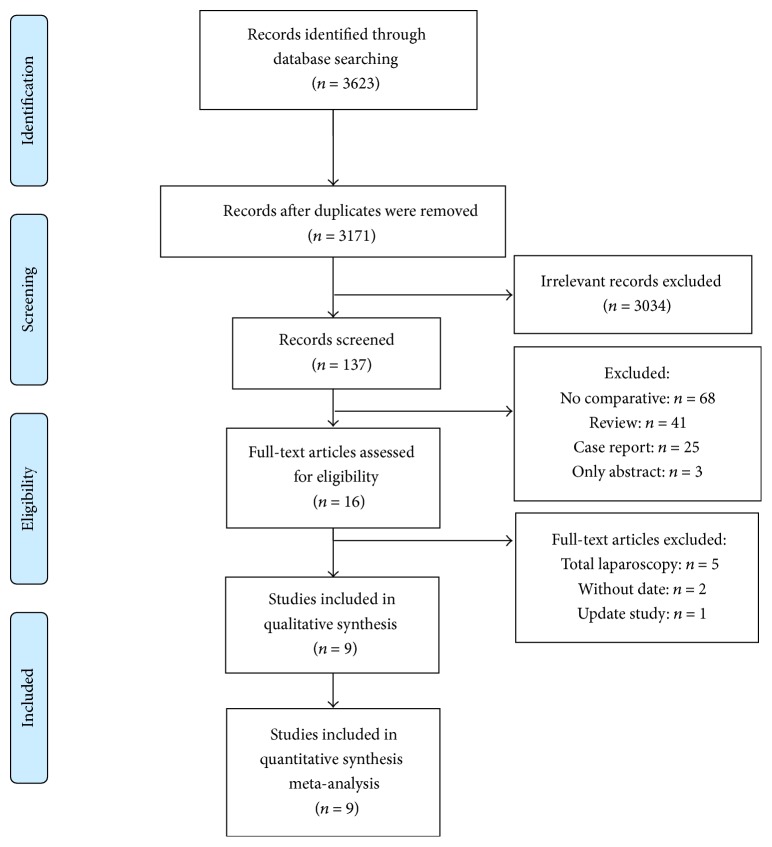
Flow diagram of included studies.

A flowchart of the search strategies, containing reasons for excluding studies, is shown in Figure 1. No randomized controlled trials were identified in the records. Nine studies were selected for the final meta-analysis. Five studies [8, 10, 12, 14, 15] compared laparoscopy-assisted and open donor right hepatectomy and one study [11] compared left hepatectomy. Two studies [5, 13] had data for both right hepatectomy and left hepatectomy comparisons. One study [9] evaluated the safety and feasibility of mixed laparoscopic-assisted donor right and left hepatectomies by comparing them with open donor hepatectomies.

A total of 979 patients were included in the analysis with 309 undergoing LADH (31.5%) and 670 undergoing OH (53.2%). Characteristics of included studies are summarized in Table 1. Four papers were conducted in Japan [5, 10, 11, 13], two in the United States [8, 9], one in China [15], one in Korea [14], and one in India [12]. Seven of the studies graded morbidity according to the Clavien-Dindo Classification. Four studies reported conversion in 10 cases, including diaphragmatic rupture (1 case), right hepatic vein injury (1 case), and IVC injury (1 case). And the other conversions were not documented in their respective studies. Three studies reported quality of life for donor in the follow-up period [11, 12, 14].

The quality of the research included was generally moderate to satisfactory. NOS shows that one out of the nine studies observed had 6 stars, six had 7 stars, and two had 8 stars.  Table 2 shows the evaluation of quality according to NOS.

### 3.2. Meta-Analysis Results

#### 3.2.1. Primary Outcome

**Figure 2:**
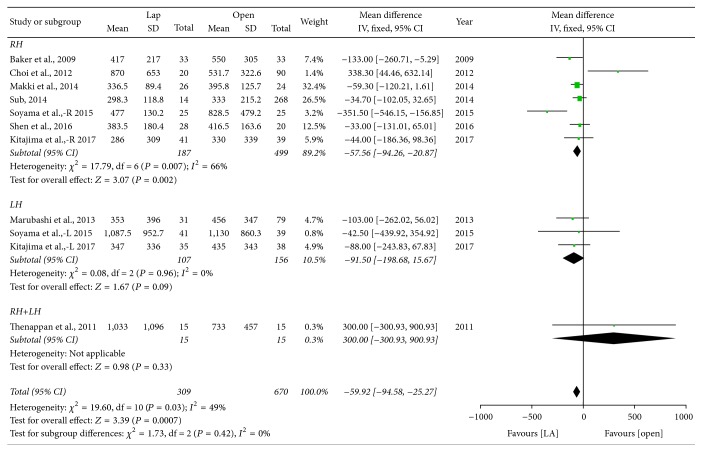
Forest plot of subgroup analyses—intraoperative blood loss. Lap: laparoscopy-assisted living donor hepatectomy, Open: open donor hepatectomy, RH: right lobe hepatectomy, LH: left lobe hepatectomy, and RH + LH: mixed group.

*Blood Loss*. Intraoperative blood loss during surgery was significantly less for laparoscopy-assisted procedures compared to open ones (WMD = −59.92 ml; 95% CI: −94.58~−25.27, *P* = 0.0007) (Figure 2). In the subgroup analysis, LADH was a protective effect against blood loss compared with ODH in RH (WMD = −57.56 ml; 95% CI: −94.26~−20.87, *P* = 0.002). For the LH group, the results also show that LADH incurred lower blood loss (WMD = −91.50 ml; 95% CI: −198.68~15.67, *P* = 0.08). Furthermore, the difference was not significant in the mixed group (WMD = 300 ml; 95% CI: −300.93~900.93, *P* = 0.33).

**Figure 3:**
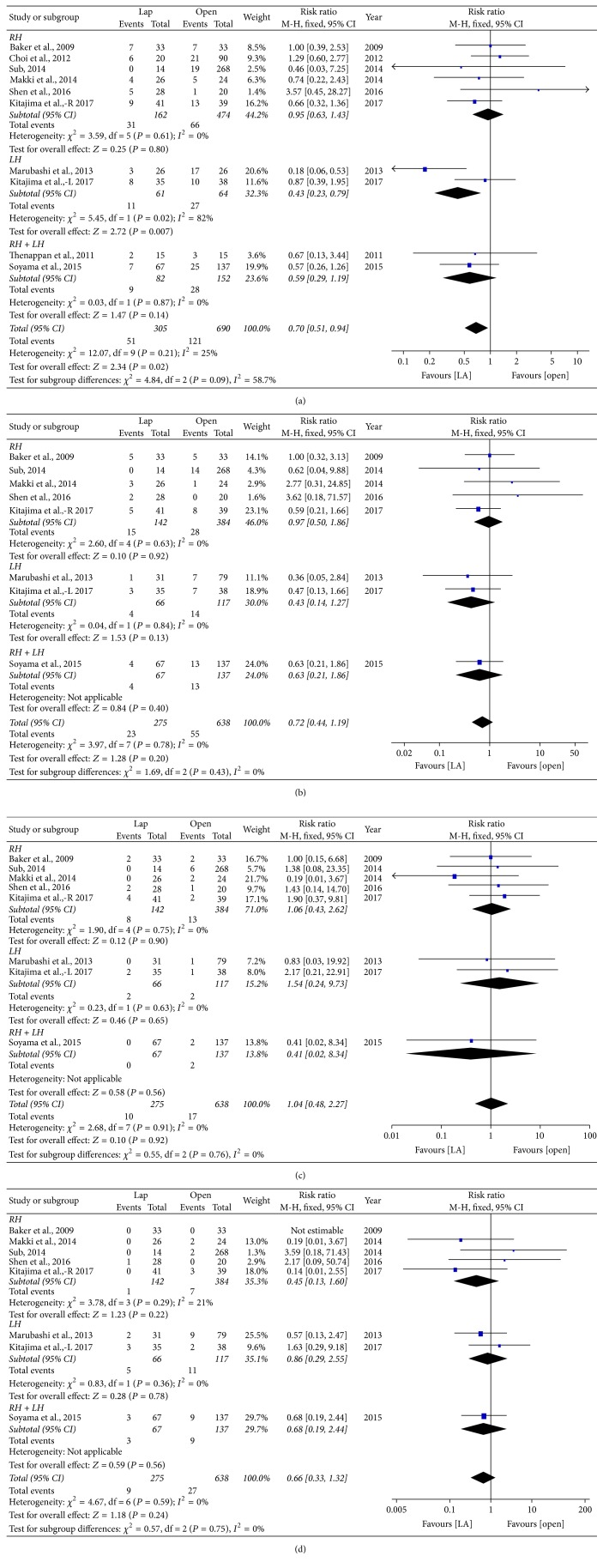
Forest plot of subgroup analyses. (a) Overall postoperative complications. (b) Clavien grade I complication. (c) Clavien grade II complication. (d) Clavien grade III complication. Lap: laparoscopy-assisted living donor hepatectomy, Open: open donor hepatectomy, RH: right lobe hepatectomy, LH: left lobe hepatectomy, and RH + LH: mixed group.

*Complication*. All of the included studies reported complication rate. A reduced postoperative complication rate was observed in the LADH group (RR = 0.70, 95% CI: 0.51~0.94, *P* = 0.02) (Figure 3(a)). In the subgroup analysis, LADH was comparable to ODH in RH group (RR = 0.95, 95% CI: 0.63~1.43, *P* = 0.80) and mixed group (RR = 0.59, 95% CI: 0.29~1.19, *P* = 0.14). However, complications were significantly decreased in LADH for LH procedures (RR = 0.43, 95% CI: 0.23~0.79, *P* = 0.007). There are no differences between the two groups regarding the Clavien grades I to IV and V complications (Figures 3(b), 3(c), and 3(d)). Postoperative complications included in this study are summarized in Table 3.

**Figure 4:**
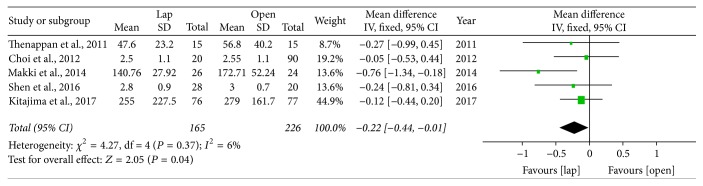
Forest plot of meta analyses—analgesic use.

*Analgesic Use*. There are five studies that gave relevant information on analgesic use after surgery and postoperative pain was evaluated by the number of days of analgesic use or the dosage of analgesic. We found that analgesic use was significantly less in the LADH group (SMD = −0.22; 95% CI: −0.44~−0.11, *P* = 0.04) (Figure 4).

#### 3.2.2. Secondary Outcomes

**Figure 5:**
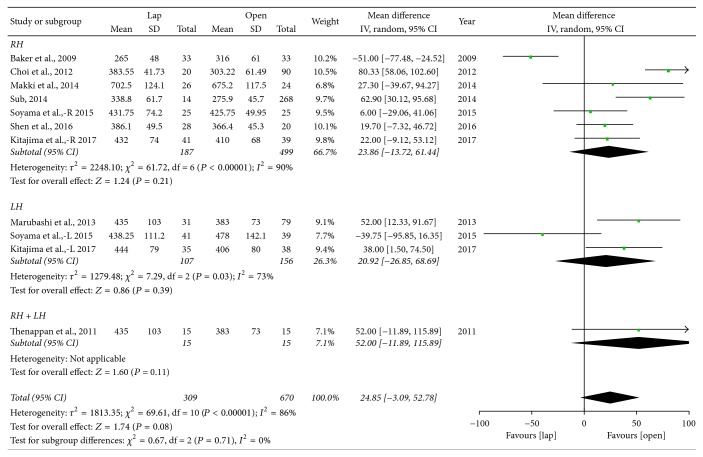
Forest plot of subgroup analyses—operation time. Lap: laparoscopy-assisted living donor hepatectomy, Open: open donor hepatectomy, RH: right lobe hepatectomy, LH: left lobe hepatectomy, and RH + LH: mixed group.

*Operative Time*. Nine of the included studies [5, 8–15] reported operation times and mean operation time tended to be longer in LADH compared to ODH (WMD = 24.85 min; 95% CI: −3.01~52.78, *P* = 0.08) (Figure 5). Two of the studies [5, 13] provided data for right lobe hepatectomy (RH) and left lobe hepatectomy (LH), respectively, and we then did a subgroup analysis of RH, LH, and mixed group. The subgroup analysis shows that there was no significant difference in operation time in LADH and ODH groups in RH (WMD = 23.86 min; 95% CI: −13.72~61.44, *P* = 0.21), LH (WMD = 20.92 min; 95% CI: −26.85~68.69, *P* = 0.39), and mixed (WMD = 52 min; 95% CI: −11.89~68.894, *P* = 0.11) subgroup.

**Figure 6:**
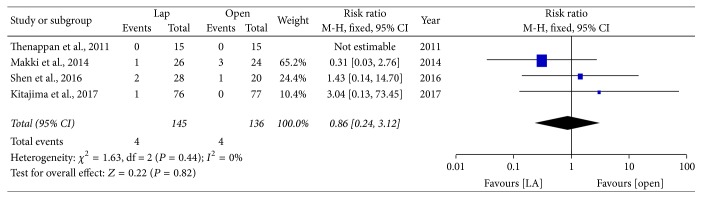
Forest plot of meta analyses—transfusion. Lap: laparoscopy-assisted living donor hepatectomy, Open: open donor hepatectomy.

*Transfusion*. Five studies reported transfusion information, with similar outcomes in both LADH and ODH (RR = 0.82; 95% CI: 0.24~3.12, *P* = 0.82) (Figure 6).

**Figure 7:**
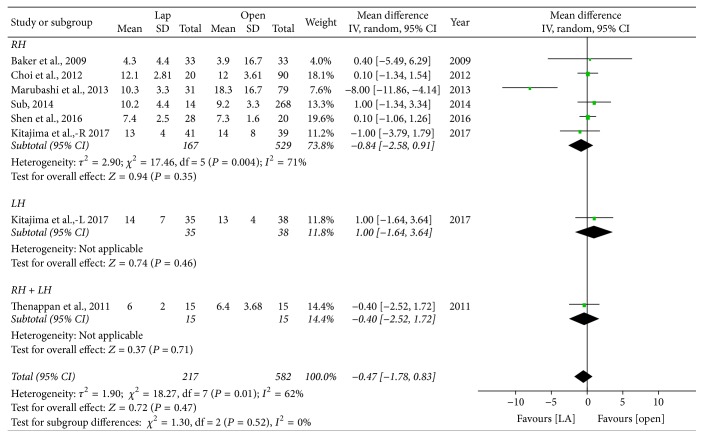
Forest plot of subgroup analyses—length of hospital stay. Lap: laparoscopy-assisted living donor hepatectomy, Open: open donor hepatectomy, RH: right lobe hepatectomy, LH: left lobe hepatectomy, and RH + LH: mixed group.

*Length of Hospital Stay*. Length of hospital stay was similar between LADH and ODH (WMD = −0.47 d; 95% CI: −1.78~0.83, *P* = 0.47) (Figure 7). For the subgroup analysis, there were no significant difference between LADH and ODH in the RH group (WMD = −0.84 d; 95% CI: −2.58~0.91, *P* = 0.35), LH (WMD = 1.00 d; 95% CI: −1.64~3.64, *P* = 0.46), or the mixed group (WMD = −0.40 d; 95% CI: −2.52~1.72, *P* = 0.71).

#### 3.2.3. Graft Weight

**Figure 8:**
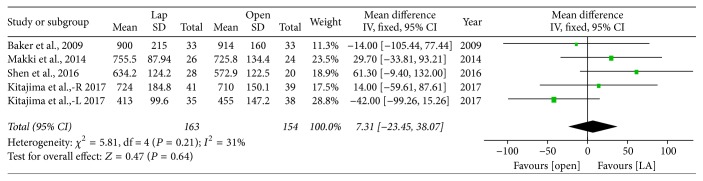
Forest plot of meta analyses—graft weight.

A total of 4 studies reported graft weight, showing no difference between the two groups (WMD = 7.31 g; 95% CI: −23.45~38.07, *P* = 0.64) (Figure 8).

#### 3.2.4. Publication Bias

**Figure 9:**
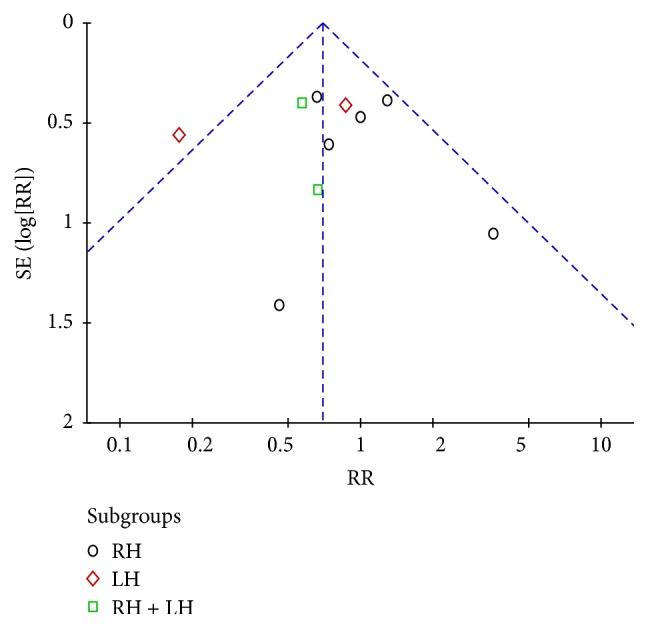
Funnel plot of overall postoperative complications. RH: right lobe hepatectomy, LH: left lobe hepatectomy, and RH + LH: mixed group.

A funnel plot for studies reporting RRs of postoperative overall complications was used to detect publication bias. The plots standing for the studies distributed symmetrically. This result suggested that the publication bias was acceptable (Figure 9).

## 4. Discussion

Minimally invasive donor surgery was developed to reduce the morbidity and decrease the impact on the donor, minimizing tissue trauma, and improving postoperative pain and cosmesis for patients. LADH with manual hand manipulation in the abdominal cavity, giving the surgeon enhanced tactile feedback of the liver, allowed for more precise mobilization and dissection of the targeted lobe. This technique is combined with smaller incision while preserving the maneuverability and safety of an open liver resection. LADH apparently leads to less wound-related morbidity and the best cosmetic result [16]. In a recent review, Xu et al. [17] examined laparoscopic versus open liver resection for liver transplantation, showing less blood loss, shortened hospital stay, and longer operation time. However, this review did not attempt to clarify the different types of laparoscopic surgery. In our meta-analysis, we only included the studies of laparoscopy-assisted (hybrid) surgery. Our further subgroup analysis was done to learn how LADH affects surgery in different areas of the liver.

Our result confirms that blood loss was significantly less in the LADH group than in the ODH group. This is consistent with published results for laparoscopic hepatectomies, even when laparoscopy is only used for the hepatic mobilization [18]. In the subgroup analysis of single types of hepatectomy to minimize the bias, there was no difference between the types of donor hepatectomy. LADH is a potential technique to decrease blood loss, confirmed by the colorectal surgery [19] and prior analysis [20]. Hand-assisted surgery has been promoted by its advocates in decreased complication rate in the colorectal surgery [19]. Our analysis of LADH demonstrated favourable overall complication rates compared to ODH, similar to the previous analysis [20]. In the subgroup analysis, LH shows a significantly lower rate of complications in the LADH group, which accounts for the lower complication rate in the total group. However, the case volume is small in the left hepatectomy subgroup. In theory, it is easier to mobilize the right lobe from the diaphragm by laparoscopic technique and inferior vena cava with the help of manual manipulation. Adequate mobilization, improved visualization, and better manipulation contribute to the enhanced safety of the operation. Living donor mortality in ODH was reported as 0.2% (23/1153), mostly related to surgical procedure [21]. There was no mortality to be reported in the studies both in laparoscopy-assisted and open group for donor. In other words, LADH shows a better tendency toward in the outcome of morbidity to ODH.

Smaller and midline incisions in the supraumbilical area resulted in reduced disruption of abdominal muscles, deceased scar discomfort, and less postoperative analgesic use in our analysis, raising the possibility of better cosmetic results and, possibly, faster return to work and normal physical activities. However, it tended to have an increased operative time associated with hand-assisted surgery, though it did not reach statistical significance. The result could be explained by the application of laparoscopic instruments for the meticulous mobilization in the liver surgery. Furthermore, the transfusion rate was comparable between LADH and ODH in this analysis. Additionally, LOS demonstrated no inferiority for LADH. Interestingly, the prior meta-analysis of laparoscopic versus open hepatectomy for live liver donor has shown the significantly shorter hospital stay in the LADH group [17, 20]. This may be ascribed to the methods of surgery and postoperation protocols and insurance policy. Regarding hospital cost, it was higher in the LADH. From published data, the overall cost of laparoscopic liver resection was lower than open liver resection [22].

After comparing laparoscopic-assisted operation and open operation, there was a high heterogeneity in the analysis, even in the subgroup analyses by type of surgery. These may result from differences in study designs, number of participants, donors' baseline characteristics, surgical techniques, and surgical types. In addition, some of the data estimated the mean and SD from median and range, which may result in inaccuracy. No random trials were included and most of the studies were cohort studies or case-control studies. Because of high-risk in the donor hepatectomy, a relative surgical abstention may present in the enrolled patients and their families. Based on these limitations, larger prospective studies and randomized trials are needed.

## 5. Conclusion

According to our data, laparoscopy-assisted living donor hepatectomy (LADH) is equally safe and effective technique. There was no increased risk of morbidity compared to ODH patients in our examined groups. Benefits of laparoscopy-assisted donor hepatectomy compared to open surgery have demonstrated improved short-term outcomes, especially lower intraoperative blood loss and complications. We conclude that LADH is an appropriate minimally invasive procedure for living donor hepatectomies, which needs to be selected by patients' and surgery' preferences.

## Supplementary Material

Supplementary material file S1: PRISMA Checklist.

## Figures and Tables

**Table 1:** Summary of studies included in the meta-analysis of laparoscopy-assisted versus open living donor hepatectomy.

Author	Region	Study design	Year	Study period	Lobe	Incision	Approach	Sample size	Age (year)	BMI	Sex (M/F)	Follow-up (month)	Parenchyma dissection	Graft weight (g)	Wound infection rate (%)	Incisional hernia rate (%)	Dindo- Clavien
Baker et al. [8]	USA	OCS (R)	2009	2006–2008	Ri	UMI	LA	33	37.0 ± 10.3	25.8 ± 4.1	15/18	3	—	900 ± 215	3.0	—	Yes
							Open	33	39.1 ± 11.1	25.9 ± 4.3	13/20		—	914 ± 160	—	—	
Thenappan et al. [9]	USA	OCS (R)	2011	2005–2009	Le, Ri	UMI	LA	15	33.9 ± 9.0	—	7/8	—	—	—	6.7	6.7	No
							Open	15	35.7 ± 8.1	—	6/9		—	—	0	13.3	
Choi et al. [10]	Japan	OCS (R)	2012	2008–2011	Ri	TI	LA	20	29.7 ± 10.1	23.6 ± 2.8	12/8	—	CUSA	—	10	0	No
							Open	90	36.8 ± 12.0	23.6 ± 2.9	58/32		CUSA	—	5.5	1.1	
Marubashi et al. [11]	Japan	OCS (P)	2013	2009–2012	Le	UMI	LA	31	35.8 ± 8.4	21.3 ± 3.6	13/18	13.9 ± 9.8	—	—	—	—	Yes
							Open	79	37.8 ± 10.1	22.6 ± 3.1	54/25		—	—	—	—	
Makki et al. [12]	India	OCS (P)	2014	2011–2013	Ri	UMI	LA	26	27.5 ± 9.4	24.2 ± 3.6	13/13	14 (6–22)	—	755.5 ± 87.9	11.5	—	Yes
							Open	24	32.4 ± 8.5	24.5 ± 4.4	18/6		—	725.8 ± 134.4	4.2	—	
Soyama et al. [13]	Japan	OCS (R)	2015	1997–2014	Le, Ri	UMI	LA	67	41 (26–65)	21.6 (16.9–29.0)	33/34	27	—	—	0	0	Yes
							Open	137	39 (19–67)	22.1 (16.4–34.7)	57/80	21–86	—	—	1.5	0	
Suh et al. [14]	Korea	OCS (P)	2014	2010–2013	Ri	TI	LA	14	24.9 ± 8.7	20.9 ± 2.9	206/62	32.6 (6.4–55.4)	—	—	0	0	Yes
							Open	268	34 ± 9.7	23.2 ± 3.0	1/13		—	—	1.1	0	
Shen et al. [15]	China	OCS (R)	2016	2011–2014	Ri	UMI	LA	28	40.4 ± 11.1	23.1 ± 1.8	15/13	—	CUSA	634.2 ± 124.2	0	0	Yes
							Open	20	38.3 ± 11.4	21.9 ± 1.9	13/7		CUSA	572.9 ± 122.5	0	0	
Kitajima et al. [5]	Japan	OCS (R)	2017	2011–2016	Le, Ri	UMI	LA	153	42 (20–67)	22.4 (16.5–28.7)	36/40	36.6 (1.4–66)	—	668 (460–1100)^*∗*^	0	0	Yes
							Open	77	43 (21–64)	22.7 (16.8–29.8)	43/34		—	655 (505–1025)^*∗*^	1.3	0	

OCS, observational clinical study; P, prospectively collected data; R, retrospectively collected data; LA: laparoscopy-assisted; O: open; Le, left lobe; Ri, right lobe; UMI, upper median incision; TI, transverse incision; CUSA, Cavitron Ultrasonic Surgical Aspirator; ^*∗*^right.

**Table 2:** Quality assessment based on the NOS for observational studies.

Author	Matched factors	Selection (out of 4)				Comparability (out of 2)	Outcomes (out of 3)			Total (out of 9)
		①	②	③	④		⑤	⑥	⑦	
Baker et al. [8]	abcdef	*∗*	*∗*	*∗*	*∗*	*∗∗*	*∗*			7
Thenappan et al. [9]	abcdef	*∗*	*∗*	*∗*	*∗*	*∗∗*	*∗*			7
Choi et al. [10]	abcdefghijkl	*∗*	*∗*	*∗*	*∗*	*∗∗*	*∗*			7
Marubashi et al. [11]	—	*∗*	*∗*	*∗*	*∗*	*∗∗*	*∗*	*∗*		8
Makki et al. [12]	abcd	*∗*	*∗*	*∗*	*∗*	*∗*	*∗*			6
Soyama et al. [13]	abcd	*∗*	*∗*	*∗*	*∗*	*∗∗*	*∗*			7
Suh et al. [14]	—	*∗*	*∗*	*∗*	*∗*	*∗*	*∗*	*∗*	*∗*	8
Shen et al. [15]	abcd	*∗*	*∗*	*∗*	*∗*	*∗∗*	*∗*			7
Kitajima et al. [5]	—	*∗*	*∗*	*∗*	*∗*	*∗∗*	*∗*			7

Factors matched between groups: a: age; b: gender; c: body mass index; d: hepatic artery anomalies; e: portal vein anomalies; f: biliary anomalies; g: ALT; h: AST; i: hemoglobin; j: prothrombin time prothrombin time; k: prothrombin rate; l: international normalized ratio.

**Table 3:** Systematic review of postoperative complications.

Author	Group	*n*	Event	Specified complications	Complication (%)			
					1	2	3	4
Baker et al. [8]	LA	33	7	Small bowel injury × 1, biloma × 1, wound infection × 1	15.2	6.1	0	0
	O	33	7	Biloma × 1, pleural effusion × 1, bowel obstruction × 1	15.2	6.1	0	0
Thenappan et al. [9]	LA	15	2	Wound infection × 1, incisional hernia × 1	—	—	—	—
	O	15	3	Biliary leakage × 1, incisional hernia × 2	—	—	—	—
Choi et al. [10]	LA	20	6	Wound complication × 2, diaphragmatic hernia × 1, pleural effusion × 2, biliary stricture × 1	—	—	—	—
	O	90	21	Wound complication × 5, ventral hernia × 1, pleural effusion × 4, bile leak × 8, bleeding × 1, portal versus thrombosis × 2	—	—	—	—
Marubashi et al. [11]	LA	31	3	—	3.2	0	6.5	0
	O	79	17	—	8.9	1.3	11.3	0
Makki et al. [12]	LA	26	4	—	11.5	0	3.8	0
	O	24	5	—	3.8	7.7	7.7	0
Soyama et al. [13]	LA	67	7	Biliary leakage × 2, postoperative bleeding × 2, bleeding of duodenal ulcer × 1, PV thrombus × 1, ileus × 1	6.0	0	4.5	0
	O	137	25	Biliary leakage × 10, pleural effusion × 2, infectious complication × 3, nerve paralysis × 2, postoperative bleeding × 1, acute pancreatitis × 1, skin necrosis × 1, gastric stasis × 4, PV thrombus × 1	9.5	1.5	6.6	0.7
Suh et al. [14]	LA	14	0	0	0	0	0	0
	O	268	22	Hyperbilirubinemia × 1, pleural effusion × 6, ileus × 5, wound seroma × 2, bleeding × 3, wound infection × 3, biliary stricture × 2	5.2	2.2	0.7	0
Shen et al. [15]	LA	28	5	Pleural effusion × 2, pulmonary infection × 1, ileus × 1, intra-abdominal hemorrhage × 1	7.1	7.1	0	0
	O	20	1	Pulmonary infection × 1	0	5	0	0
Kitajima et al. [5]	LA	76	17	Wound dehiscence × 2, intra-abdominal fluid collection × 4; hyperbilirubinemia × 1, fever × 2, renal failure × 1, small bowel obstruction × 1, atelectasis × 1, pleural effusion × 2, bile leakage × 3	10.5	7.9	3.9	0
	O	77	23	Wound dehiscence × 5, pleural effusion × 2, ascites, × 1, portal venous thrombosis × 3, bile leakage × 5, drug-induced hepatotoxicity × 5, intraabdominal fluid collection × 1	19.5	3.9	6.5	0

LA: laparoscopy-assisted living donor hepatectomy; open: open living donor hepatectomy.

## Abbreviations

LADH: Laparoscopy-assisted living donor hepatectomy
ODH: Open donor hepatectomy
LLR: Laparoscopic liver resection
WMD: Weighted mean difference
SMD: Standard mean difference
RR: Risk ratio
SD: Standard deviation
NOS: Newcastle-Ottawa Quality Assessment Scale
RH: Right lobe hepatectomy
LH: Left lobe hepatectomy
ALT: Alanine aminotransferase
AST: Aspartate aminotransferase
TB: Total bilirubin
LFT: Liver function test.
